# Nonpharmacological interventions for cognitive impairments following primary progressive aphasia: a systematic review of the literature

**DOI:** 10.1590/S1980-57642013DN70100018

**Published:** 2013

**Authors:** Maria Teresa Carthery-Goulart, Amanda da Costa da Silveira, Thais Helena Machado, Leticia Lessa Mansur, Maria Alice de Mattos Pimenta Parente, Mirna Lie Hosogi Senaha, Sonia Maria Dozzi Brucki, Ricardo Nitrini

**Affiliations:** 1 Núcleo de Cognição e Sistemas Complexos e Centro de Matemática, Computação e Cognição da Universidade Federal do ABC, Santo André SP, Brazil. Grupo de Neurologia Cognitiva e do Comportamento, Departamento de Neurologia, Hospital das Clínicas da Faculdade de Medicina da Universidade de São Paulo, São Paulo SP, Brazil.; 2 Núcleo de Cognição e Sistemas Complexos e Centro de Matemática, Computação e Cognição da Universidade Federal do ABC, Santo André SP, Brazil.; 3 Grupo de Neurologia Cognitiva e do Comportamento da Faculdade de Medicina da Universidade Federal de Minas Gerais, Belo Horizonte MG, Brazil.; 4 Curso de Fonoaudiologia da Faculdade de Medicina da Universidade de São Paulo, São Paulo SP, Brazil.; 5 Grupo de Neurologia Cognitiva e do Comportamento, Departamento de Neurologia, Hospital das Clínicas da Faculdade de Medicina da Universidade de São Paulo, São Paulo SP, Brazil.

**Keywords:** primary progressive aphasia, treatment, speech and language therapy, intervention, cognitive rehabilitation

## Abstract

This study provided a systematic review on nonpharmacological interventions applied to patients diagnosed with Primary Progressive Aphasia (PPA) and its variants: Semantic (SPPA), Nonfluent (NFPPA) and Logopenic (LPPA) to establish evidence-based recommendations for the clinical practice of cognitive rehabilitation for these patients.

**METHODS:**

A PubMed and LILACS literature search with no time restriction was conducted with the keywords *PPA* (and its variants) AND *rehabilitation* OR *training* OR *intervention* OR *therapy* OR *treatment* OR *effectiveness*. To develop its evidence-based recommendations, a research committee identified questions to be addressed and determined the level of evidence for each study according to published criteria (Cicerone et al., 2000). Overall evidence for treatments was summarized and recommendations were derived.

**RESULTS:**

Our search retrieved articles published from 1995 to 2013: 21 for SPPA, 8 for NFPPA, 3 for LPPA and 8 for PPA with no specification. Thirty-five studies were rated as Class III, consisting of studies with results obtained from one or more single-cases and that used appropriate single-subject methods with adequate quantification and analysis of results. The level of evidence of three functional interventions could not be established. One study was rated as Class II and consisted of a nonrandomized case-control investigation.

**CONCLUSION:**

Positive results were reported in all reviewed studies. However, in order to be recommended, some investigation regarding the intervention efficacy was required. Results of the present review allows for recommendation of some nonpharmacological interventions for cognitive deficits following PPA as Practice Options. Suggestions for further studies on PPA interventions and future research are discussed.

## Abstract (pt)

Este estudo ofereceu uma revisão sistemática de intervenções não-farmacológicas aplicadas a pacientes com Afasia Progressiva Primária (APP) e suas variantes: Semântica (APPS), Não-fluente (APPNF) e Logopênica (APPL) com o objetivo de estabelecer recomendações baseadas em evidências para a prática clínica de reabilitação cognitiva para estes pacientes.

**MÉTODOS:**

Conduziu-se busca por literatura, sem restrição de período, no PubMed e LILACS com as palavras-chave *PPA* (e variantes) AND *rehabilitation* OR *training* OR *intervention* OR *therapy* OR *treatment* OR *effectiveness*. Para desenvolver recomendações baseadas em evidências um comitê de pesquisadores identificou as questões relevantes para investigação e determinou o nível de evidência para cada estudo de acordo com critérios publicados (Cicerone et al., 2000). A evidência total para os tratamentos foi sumarizada e recomendações redigidas.

**RESULTADOS:**

Foram encontrados artigos publicados de 1995 a 2013: 21 para APPS, 8 para APPNF, 3 para APPL e 8 para APP sem especificações. Trinta e cinco estudos foram classificados como Classe III, consistindo de resultados obtidos com um ou mais indivíduos e utilizando metodologia apropriada para estudo de casos. O nível de evidência de três intervenções funcionais não pôde ser estabelecido. Um estudo foi classificado como Classe II e consistiu de pesquisa caso-controle não-randomizada.

**CONCLUSÃO:**

Resultados positivos foram reportados em todos os trabalhos analisados mas, para serem recomendados, os tratamentos requerem investigação em relação a sua eficácia. Resultados da presente revisão permitiram recomendação de algumas intervenções como Opções Práticas. Ademais, apresentamos sugestões para futuros estudos de intervenção em APP.

## INTRODUCTION

The term Primary Progressive Aphasia (PPA) was first used by Mesulam^1,2^ in order to designate a progressive and circumscribed language disorder (aphasia) with relative preservation of functioning in activities of daily living and in the absence of deficits on other cognitive domains in the first two years post-symptoms onset. Cases of PPA were generally categorized as Nonfluent Progressive Aphasia or Semantic Dementia according to the consensus of Neary et al.^3^ or as fluent and nonfluent progressive aphasia^2^ in many studies conducted since then. A third syndrome, logopenic aphasia was reported in 2004.4 The past three decades have seen a clear advance in the characterization of these syndromes with detailed descriptions of prominent speech and language deficits, regions of brain atrophy/ hypometabolism and also specification of the underlying pathology in many cases. In 2011, an international group of PPA investigators agreed on diagnostic criteria for PPA and on clinical, imaging-supported and definite pathology criteria for the diagnosis of three distinct variants: non-fluent/agrammatic (NFPPA), semantic (SPPA) and logopenic (LPPA).^5^

In a considerable number of patients with PPA the onset of the disease occurs at a young age and has a devastating effect on their functional status and quality of life. The extensive progress in PPA diagnosis has led to a growing number of patients in need of treatment alternatives. In the absence of clearly effective pharmacological options,^6^ there has been increasing interest in other approaches, particularly behavioral interventions. Croot et al. (2009) performed a broad literature review on clinical management in PPA whose main objective was to assist clinicians to make choices about speech pathology service provision. The authors reviewed 25 studies and made important considerations about intervention features and their results as well as suggestions for future research in this area. However, to our knowledge, no systematic review on nonpharmacological interventions has been conducted thus far. In addition, several studies have emerged since 2009 including biomarkers and more rigorous experimental control to measure treatment effects. Therefore, our objective was to conduct a systematic review on nonpharmacological interventions applied to patients diagnosed with PPA syndromes aimed at establishing evidence-based recommendations for the clinical practice of cognitive rehabilitation for patients with PPA. Specifically, we considered the evidence-based practice guidelines provided by the ASHA^7^ and the evidence classification criteria for cognitive rehabilitation established by Cicerone et al.^8^.

According to the International Classification of Functioning, Disability and Health (ICF)^9^ the term disability covers impairments, activity limitations and participation restrictions. Nonpharmacological interventions can focus on any of these levels. In the present paper, we classify treatments into impairment-directed and functional interventions. The former target remediation or focus on slowing the progression of specific speech and language impairments, such as naming deficits, dysgraphia, agrammatism and apraxia of speech, whereas the latter focus on functional communication including environmental modifications, compensatory strategies or increasing levels of participation in communication activities.

In 1995, McNeil et al.^10^ published a study combining pharmacological and non-pharmacological treatment for a patient with PPA. They found equivalent results for the pharmacological plus behavioral treatment compared to the provision of behavioral treatment alone. Almost 20 years on, cognitive rehabilitation in PPA is still considered a "new" area in Neuropsychology and Speech and Language Therapy with many unanswered questions. There is no consensus on recommendation criteria of different types of interventions, intensity, duration and periodicity of treatments. In addition, therapy gains concerning both evolution of cognitive symptoms, functioning in activities of daily living and quality of life of patients, their carers and family still need further investigation.

It is crucial to analyze critically the accumulated knowledge in this area to provide guidelines for future research that may increase the level of evidence about these interventions and support treatment choices in a clinical context. Moreover, we intend this paper to provide a summary and an update of recent findings for therapists practicing this area.

## METHOD

In order to carry out a systematic review that would encompass international as well as Latin-American studies, two indexing databases were consulted: PubMed, from the National Library of Medicine of the United States of America, and LILACS, the Latin American and Caribbean Health Sciences Literature database. The terms entered in both databases were the following: [1] "Primary Progressive Aphasia AND (rehabilitation OR training OR intervention OR therapy OR treatment OR effectiveness)", [2] "Semantic Dementia AND (rehabilitation OR training OR intervention OR therapy OR treatment OR effectiveness)", [3] "Nonfluent Progressive Aphasia AND (rehabilitation OR training OR intervention OR therapy OR treatment OR effectiveness)", [4] "Logopenic aphasia AND (rehabilitation OR training OR intervention OR therapy OR treatment OR effectiveness)". The retrieved titles were submitted to the following exclusion criteria: Titles that were clearly not about PPA, papers that were not written in English, French, Portuguese or Spanish were all excluded. Subsequently, abstracts of the selected titles were read, and the following exclusion criteria were applied: review studies were excluded, except for those that mentioned treatments and interventions for Frontotemporal Lobar Degeneration syndromes. All papers that made no mention of any type of non-pharmacological treatment were also excluded. Case studies were kept for further analysis, since they may mention a nonpharmacological treatment undertaken by the patient in the body of the text. After abstract selection, their respective articles were read. References of selected papers were also scanned in order to identify other related papers that were not indexed in the searched databases but would also contribute to this review. The same exclusion criteria mentioned before were applied to the titles found in the references of the selected papers. Only complete manuscripts published in indexed journals were included, therefore interventions published as book chapters and conference abstracts were not analyzed.

This selection procedure was performed by three of the authors of this study, so as to ensure an acceptable degree of agreement. To develop its evidence-based recommendations, a research committee identified questions to be addressed and determined the level of evidence for each study according to published criteria for cognitive rehabilitation.^8^ After reading the papers, the authors agreed on the following variables to be observed and used as classifying criteria in the studies: diagnosis, duration of the disease at intervention, age at intervention, sex, educational level, study design, intervention type, intervention features (goals, procedures, language spoken, individual/group sessions, intervention length, frequency and duration of sessions, home-practice, involvement of a caregiver, materials), outcome measures, results, maintenance of gains, generalization, follow-up and comparison of measures of brain activity pre and post-treatment. Overall evidence for treatments for each PPA subtype was summarized and recommendations were derived from consideration of the strengths of evidence. The main features of the studies are summarized in the tables presented in the results section of this paper.

## RESULTS

Searches on PubMed and LILACS databases retrieved 814 and three titles, respectively. More specifically, combined with "(rehabilitation OR training OR intervention OR therapy OR treatment OR effectiveness)", the term "Semantic dementia" retrieved 537 titles, "Non-fluent progressive aphasia", 133, "Primary progressive aphasia" 124, and "Logopenic aphasia", 20 titles on PubMed. The three titles found on LILACS were all retrieved with the term "Primary progressive aphasia", whereas all other combinations retrieved no titles. Many of the search results overlapped, and after applying the exclusion criteria, only one article from LILACS remained, whereas 19 articles from PubMed were selected. Scanning the references from these selected papers, and also references from case studies and review articles previously found on PubMed and LILACS, retrieved another 19 papers, which were added to the final list in order to be analyzed. In summary, this paper analyzed a total of 39 articles related to interventions on PPA. The search took place on November of 2012 and was repeated in January 2013. The complete list of selected papers is found in Tables 1-4. Detailed information including study design, description of interventions, pre and post-assessment tools, and results is provided in Appendix 1, available when this manuscript is accessed online from the Dementia & Neuropsychologia site (at www.demneuropsy.com.br).

**Table 1:** Intervention studies in Semantic Variant PPA.

	Studies grouped by type of intervention	Characteristics of participant(s) Age (years), sex, education, disease duration	Intervention goals
**Impairment-directed interventions**	Graham et al. (1999; 2001)^11,12^	69; male; Doctorate; 4 years	Naming and lexical retrieval
	Snowden, Neary (2002)^13^	61; female; N/A; N/A54; female; N/A; N/A	Naming
	Bozeat, Patterson, Hodges (2004)^14^	58; female; N/A; 3 years	Object use
	Frattali (2004)^15^	66; male; Higher education; N/A	Naming
	Jokel, Rochon, Leonard (2002; 2006)^16,17^	63; female; Bachelor 's; 7 years	Naming
	Bier et al. (2009)^18^	70; female; High school; 5 years	Concept relearning (Naming and semantic attributes)
	Dewar et al. (2009)^19^	63; male; Bachelor 's; 4 years	Naming and learning semantic attributes
	Heredia et al. (2009)^20^	53; female; Well-educated civil servant; 2 years	Naming
	Newhart et al. (2009)^21^	60; female; Master 's; N/A	Naming and lexical retrieval
	Robinson et al. (2009)^22^	63; female; Some college; 3 years63; female; N/A; 3 years	Naming, definition and object use
	Dressel et al. (2010)^23^	48; male; College; 2 years	Naming
	Jokel, Rochon, Anderson (2010)^24^	N/A; male; Bachelor 's; 2 years	Naming
	Montagut et al. (2010)^25^	68; male; Elementary; 7 years	Naming and lexical retrieval
	Senaha, Brucki, Nitrini (2010)^26^	55; female; Some college; 2 years77; male; Bachelor 's; 1 year56; male; Bachelor 's; 2 years	Naming and lexical retrieval
	Mayberry et al. (2011)^27^	65; female; N/A; 4 years53; male; N/A; 4.5 years	Naming
	Jokel, Anderson (2012)^28^	From 56 to 87; 3 males and 4 females; from high school to Master 's degree; from 2 to 6 years	Naming
	Savage et al. (2012)^29^	From 54 to 69; 4 males; Some college; from 4 to 5 years	Naming and lexical retrieval
**Functional interventions**	Wong et al. (2009)^30^	63; male; 14 years; 2 years	Communication effectiveness: improvement/maintenance of discursive skills
	Bier et al. (2011)^31^	68; female; Bachelor 's; 4 years	Learning semantic attributes/ activity participation rehabilitation

**Table 2:** Intervention studies in Nonfluent / Agrammatic Variant PPA.

	Studies grouped by type of intervention	Characteristics of participant(s)Age (years), sex, education, disease duration	Intervention goals
**Impairment-directed interventions**	Schneider, Thompson, Luring (1996)^32^	62; female; Some college; 2.5 years	Agrammatism
	Louis et al. (2001)^33^	64; female; N/A; N/A71; female; N/A; N/A77; male; N/A; N/A	Phonological skills
	Jokel et al. (2009)^34^	58; female; Bachelor 's; N/A75; female; Bachelor 's; N/A	Naming and lexical retrieval
	Marcotte, Ansaldo (2010)^35^	60; male; Professional; 2 years	Naming
	Henry et al. (2013)^36^	73; female; Professional; 5 years	Speech production (apraxia of speech)
**Functional interventions**	Murray (1998)^37^	64; female; High school; 4 years	Auditory and reading skills/ Communicative skills
	Rogers, Alarcon (1999)^38^	69; male; Master 's; 4 years	Communicative skills
	Pattee, Von Berg, Ghezzi (2006)^39^	57; female; N/A; 5 years	Communicative skills

**Table 3:** Intervention studies in Logopenic Variant PPA.

	Studies grouped by type of intervention	Characteristics of participant(s) Age (years), sex, education, disease duration	Intervention goals
**Impairment-directed interventions**	Newhart et al. (2009)^21^	65; female; Master 's; N/A	Naming and lexical retrieval
	Beeson et al. (2011)^40^	77; male; Professional; 2.5 years	Naming and lexical retrieval
	Tsapkini, Hillis (2013)^41^	62; female; Bachelor 's; 6 years	Spelling

**Table 4:** Intervention studies in PPA.

	Studies grouped by type of intervention	Characteristics of participant(s) and further information on PPA	Intervention goals
**Impairment-directed interventions**	McNeil, Small, Masterson, Fossett (1995)^10^	61; male; N/A; 9 months (no further information about patient's impairment was given)	Lexical semantic retrieval
	Finocchiaro et al. (2006)^42^	60; male; N/A; N/A	Naming and lexical retrieval
	Henry, Beeson, Rapcsak (2008)^43^	N/A; N/A; N/A; 5 years(fluent with characteristics towards non- fluent aphasia, incl. mild agrammatism, phonemic paraphasias, and apraxia of speech)N/A; N/A; N/A; 6 years(fluent aphasia, surface dysgraphia)	Naming and lexical retrieval
	Rapp, Glucroft (2009)^44^	55; female; College; 9 years (dysgraphia)	Dysgraphia
	Snowden et al. (2012)^45^	60; male; Academic; 2 years	Facilitating access to letter names and sounds (to assist reading words aloud)
**Functional interventions**	Cress, King (1999)^46^	59; female; N/A; 5 years60; male; Doctorate; 7 yearsFor both cases, MRI revealed atrophy of the left temporal lobe, and defined a diagnosis of PPA without dementia	Communication, comprehension and expression
	Cartwright, Elliott (2009)^47^	From 59 to 66; Tertiary education; 4 PPA (3 nonfluent aphasic women, 1 man with dense semantic deficits); N/A	Enhancing participant's access to TV content
	Farrajota et al. (2012)^48^	68 (mean); 11.6 years (mean); 3 years (mean); N/A10 patients (2NFPPA, 2SAPPA, 6LPPA)	Ability to communicate by verbal means in everyday life situations

Interventions were grouped first by diagnosis and then intervention type (impairment-directed vs. functional, similarly to Croot et al.9). Thus, Table 1 summarizes findings for SPPA, with 21 papers revised (19 impairment-directed and two functional interventions). Table 2, NFPPA, lists eight papers, five impairment-directed and three functional interventions. Table 3, LPPA, contains three manuscripts reporting impairment-directed interventions. And finally, Table 4 is for PPA, including studies that have not been classified into PPA subtypes or studies that analyzed groups of PPA patients with no concern for specific variants or that included patients that do not conform to the prototypes defined in the international consensus. Eight papers were included in Table 4, five impairment-directed interventions and three functional interventions.

Papers regarding SPPA interventions (Table 1) were published between 1999 and 2012, most of them were case reports of a maximum of four patients. The mean age of patients described in treatments was 62.48 years old (SD=8.50, range 53-87) at intervention baseline, 52% were men, and patients reported an average disease duration of 3.6 years (SD=1.55). In general, therapies varied from one single session to 18 months for impairment-directed therapies, and from five to 48 months for the functional interventions. Six interventions were exclusively based on home practice, where therapies at home varied from three to 10 weeks. From 2009 onwards, strategies that included pictures began to include not only presentation on paper cards, but also on the computer screen. Except for two, all interventions included follow-up that ranged from two weeks to two years. Only one study addressed post-intervention changes in brain activity^23^ and similarly only one calculated effect sizes for therapy results.^29^ Twenty studies were rated as Class III, consisting of results obtained from one or more single-cases and that used appropriate single-subject methods with adequate quantification and analysis of results. The level of evidence of the functional intervention carried out by Wong et al.^30^ could not be established due to absence of reliable methodological control to determine treatment effects.

NFPPA papers (Table 2) were published between 1996 and 2013 and described patients whose mean age was 66.45 years old (SD=6.96, ranging from 58 to 77 years). 72% of the cases were women, varying from one to three patients. On average, impairment-directed interventions had around 22 sessions (SD=20.57), ranging from 8 to 60 training sessions. When home-practice sessions were not taken into account for calculation, the average number of sessions was 10.6 (SD=3.43). Only three studies reported follow-up testing, which ranged from one to 12 months. One study reported fMRI investigation supporting therapy results.^35^ Functional interventions presented longer durations, from 9 weeks to 4 years of therapy. Seven studies were rated as Class III, consisting of results obtained from one or more single-cases and that used appropriate single-subject methods with adequate quantification and analysis of results. The level of evidence of the functional intervention carried out by Rogers & Alarcon^38^ could not be established due to absence of reliable methodological control to determine treatment effects.

Regarding LPPA interventions (Table 3), papers were published between 2009 and 2013 and the reported cases presented a mean age of 68 years old (SD=7.93). Therapy duration ranged from two to 11 weeks and home practice and was emphasized in only one study^40^ which was also the only study that included follow-up testing (six months after intervention). Two interventions aimed to treat naming and lexical retrieval deficits and one targeted spelling deficits. Only one study reported use of cerebral imaging data (fMRI) as a post-treatment measure.^40^ The three studies were rated as Class III, consisting of results obtained from one or more single-cases and that used appropriate single-subject methods with adequate quantification and analysis of results.

The PPA table (Table 4) summarized eight papers published from 1995 to 2013, including a total of 21 cases whose mean age was 60.88 years old (SD=3.88, ranging from 55 to 68 years). Patients' clinical features varied in these studies, as did intervention goals. One of these studies employed high-frequency repetitive Transcranial Magnetic Stimulation.^42^ Half of the studies entailed follow-up testing, which ranged from one month up to 3 years. One study reported the effect size of the intervention.^43^ Six studies were rated as Class III, consisting of results obtained from one or more single-cases and that used appropriate single-subject methods with adequate quantification and analysis of results. The level of evidence of one functional intervention 46 could not be established due to absence of reliable methodological control to determine treatment effects. One study was rated as Class II and consisted of a nonrandomized case-control investigation.^48^

Overall, among the thirty-nine selected manuscripts, thirty-five studies were rated as Class III and one study was rated as Class II. The level of evidence of three functional interventions could not be established due to absence of reliable methodological control to determine treatment effects.

## DISCUSSION

In the present study, we conducted a systematic review of the literature aimed at establishing evidence-based recommendations for the clinical practice of cognitive rehabilitation for patients with PPA. In order to achieve this, we summarized and examined the accumulated knowledge concerning non-pharmacological treatments for patients with PPA. We deliberately chose not to restrict our search to a specific period of time or to define strict inclusion and exclusion criteria for the studies in order to gather as many reports as possible. To accomplish this, we singled out papers from two widely used databases and followed up all relevant references cited in the selected manuscripts. Even using very inclusive criteria we were able to report only 39 studies which described treatments applied to 67 patients to date. This lack of studies was found to be even more critical when we analyzed treatment alternatives according to specific PPA subtypes. This analysis revealed the scarcity of reports for NFPPA and LPPA cases.

We organized this section under five topics. Firstly, we discuss separately the main research findings for SPPA, NFPPA and LPPA variants. Considerations were then made concerning treatments targeting patients whose impairments either do not conform to the above-mentioned prototypical syndromes, or were not classified according to the 2011 international consensus, or treatments directed to a group of PPA patients with no special concern about different subtypes. We then made our concluding comments and evidence-based recommendations for the clinical practice of cognitive rehabilitation for patients with PPA.

**Non-pharmacological interventions for patients with semantic variant PPA.** We found descriptions of treatments applied to 33 patients with SPPA.

Functional interventions targeted communication effectiveness through improvement or maintenance of discursive skills^30^ or used an ecological-approach aimed at increasing participation in meal preparation.^31^ The strength of these proposals is that they focused on the patients' needs and try to establish a direct link between therapy practices and performance in daily routine tasks. Their weakness is the difficulty achieving the necessary experimental control to measure treatment effects. In their pilot study, Bier et al.^31^ provided a good example of how this can be achieved in future research. Despite several limitations imposed by the patient's personality, authors were able to employ ABA design (baseline condition followed by treatment and then returning to baseline) and include control tasks and quantitative measures to determine therapy gains.

Most impairment-directed interventions targeted picture naming skills and lexical retrieval. Only two studies addressed face-name associations^19,26^ and another two addressed object use.^14,22^ It has been shown consistently across studies that SPPA patients are able to relearn target vocabulary during the active phase of treatment and to maintain gains above baseline levels for variable periods after ceasing intervention. This last point, however, needs to be further investigated since differences in study design and patients' profile (demographic, neuropsychological and disease duration) preclude drawing conclusions on how long therapy gains are maintained.

Another point of concern is generalization of learning to untrained stimuli or even to the same stimuli presented in a different context. Overall, this has not been achieved (e.g. Snowden and Neary13) with a few exceptions.^19,24,25,27,28^ This aspect should be a point of concern when selecting the set of stimuli to be trained and may also suggest the need for more context-based interventions. Recent studies have tried to fulfill this need by using personalized materials such as digital photos of individual household items^29^ or by training relevant activities for the patient such as cooking, as in the study of Bier et al.^31^ The impact of interventions on connected speech measures or quality of life has not been consistently investigated in studies and when reported have shown modest results.^19,20,28^ Future studies should address functional communication measures such as analysis of discursive skills to reliably establish transference of therapy gains.

Regarding learning mechanisms, studies have shown that patients relearn significantly more items when they retain residual semantic knowledge about them^13,17,28^ and are able to link them to personal experience and context.^13^ This is said to be due to overreliance on the hippocampus and adjacent medial temporal lobe structures (episodic memory system) for learning verbal labels and no reliance on temporal anterior lobe structures (affected by the disease) crucial for semantic generalization. This view has been recently challenged.^27^ By careful selection of foils the authors showed that patients used verbal labels incorrectly for foils visually and semantically associated with the target (overgeneralizations) but not for other types of foils and suggested that the impaired neocortex also plays a role in SPPA relearning skills. Overall, these findings have practical implications and suggest that therapy benefits are maximized if interventions start as early as possible in the presence of very mild semantic memory deficits and low levels of brain atrophy.

Recent studies experimentally addressed important questions concerning treatment duration and intervention strategies. It has been shown that longer therapies are more effective in the maintenance of gains than shorter ones,^29^ errorless learning is more effective than errorful learning, but same gains are achieved for active and passive learning,^28^ simple repetition of verbal label leads to similar gains to spaced-retrieval techniques,^18^ and simple picture-word matching leads to similar gains to sentence generation and to providing definitions for specific items.^29^ Nevertheless, all these issues need replication in future studies.

It is interesting to note that learning was achieved in SPPA patients after interventions of a single session;^14^ individual home-practice,^11,12^ and computer-based therapies.^24,18^ Future studies should compare these treatments to long-term interventions delivered by a therapist and also to combined interventions, in order to characterize suitable patients for each therapy type. Individual home-practice, especially aided by a computer,^31^ seems to be a very promising alternative both economically and also in terms of reducing caregivers' load.

Finally, the only study that addressed changes in brain activity after behavioral interventions in SPPA^23^ suggested that patients engage unimpaired structures such as the right superior and inferior temporal gyrus to compensate for the brain damage. This finding needs to be explored in more depth by future studies to confirm these results.

**Non-pharmacological interventions for patients with NFPPA variant.** This paper presents the results of non-pharmacological treatment applied to 11 patients in eight articles. With a few exceptions,^33,34^ most studies consisted of analyses of treatments applied to a single case.

All three studies that reported functional interventions used Augmentative and Alternative Communication (AAC) devices.^37-39^ Although positive results in communication were reported with these interventions, some patients may show reluctance to use these tools. For instance, the patient studied by Pattee et al.^39^ preferred American Sign Language and mentioned she did not feel "normal" using the digitized speech device introduced in the intervention. Moreover, successful long-term therapies using AAC devices should be described in more detail to be replicated with other patients. A study described a 4-year intervention without a clear description of the procedures, therefore rendering it difficult to reproduce.^38^

Impairment-directed interventions aimed at different aspects in NFPPA: agrammatism,^32^ phonological skills,^33^ apraxia of speech^36^ and naming and lexical retrieval deficits.^34,35^ Unlike SPPA, most NFPPA patients presented generalization of therapy gains to some extent, verified in untreated items,^32,34^ different tasks^33,34^ or functional communication evaluated through self-ratings of treatment effects.^36^ These results are encouraging but need to be replicated in a larger number of patients.

In this variant subtype, only one study evaluated treatment gains through imaging methods. Using fMRI, Marcotte and Ansaldo^35^ proposed that adaptive brain plasticity operates differently in NFPPA and post-stroke lesions, both for spontaneous recovery and therapy-induced effects. Spontaneous recovery in NFPPA involved bilateral compensation whereas in the post-stroke patient the right hemisphere was recruited. After a therapy involving the semantic approach, the authors observed an expansion of networks involving semantic processing areas (i.e., left middle and superior temporal gyrus and inferior parietal lobe bilaterally) in the patient with NFPPA, whereas in the patient with stroke, a contraction of the network occurred involving phonological processing and speech programming areas. These findings imply different brain plasticity mechanisms for aphasia depending on etiology (reorganization post-stroke vs. reactivation in NFPPA) and must be readdressed in future research.

**Non-pharmacological interventions for patients with LPPA.** As LPPA has been only recently described,^4^ it is unsurprising that the number of intervention studies with this subtype is still limited. Only three single-case studies were found, all describing impairment-directed interventions designed to improve naming/lexical retrieval and spelling. In addition, we found no studies focusing on discursive abilities. One possible explanation is that multi-modalities cognitive training directed for the mixed symptoms of this subtype, linking executive and working memory to discursive abilities, have yet to be developed.

The three studies found showed significant treatment effects for trained items while two also demonstrated generalization to untrained items^21,40^ and also to conversation skills.^40^ It is important to consider that the study which showed limited results^41^ involved a patient with six years of disease duration at intervention, a possible explanation for the unremarkable results observed.

Regarding intervention procedures, Beeson et al.^40^ attributed the success of their intervention to the approach employed which was active, errorful and intensive, involving problem-solving and generation of semantic information to facilitate lexical-retrieval. This hypothesis must be addressed empirically (as in the study of Jokel et al.^28^ with SPPA patients) and, if confirmed, suggests that naming deficits should be treated with different approaches according to PPA subtypes.

Lastly, post-treatment fMRI activation changes in an LPPA patient suggest that the behavioral improvements are supported by increased reliance on the left prefrontal cortex during word retrieval, thus recruiting relatively unimpaired networks as compensatory mechanisms.^40^ As previously mentioned for studies on the SPPA variant, this finding must be further explored in future studies.

**Non-pharmacological interventions for patients with PPA.** We identified eight studies that reported interventions for 21 patients with PPA. Again, most studies reported treatments offered to one (five studies) or fewer patients (two studies) with no special concern about a specific PPA subtype. One exception in this section, and in the entire review, is the study of Farrajota et al.^48^ in which speech-therapy gains were studied by comparing two groups of patients with PPA, one receiving treatment and another not. This is the first study designed with a control-group of PPA participants, matched by age, education and language deterioration measures. The authors used the international consensus classification but did not take subtypes into consideration when analyzing their results. This poses some limitations to the interpretation of the findings (i.e. treatment resulted in better naming skills post-treatment but group not receiving treatment comprised more SPPA patients, tending to have more severe naming deficits.^49^

Two studies included patients with atypical profiles and described effective interventions for dysgraphia^44,45^. The need for detailed assessment to design personalized interventions directed to specific cognitive deficits is emphasized.^45^ Another promising alternative for PPA is high-frequency repetitive Transcranial Magnetic Stimulation^42^ and ecological approaches such as aphasia-friendly TV viewing.^47^ Both interventions showed positive results but need replication in a larger number of patients.

**Final considerations.** We conclude this systematic review attempting to answer the following questions: [1] Can treatment be beneficial for patients with PPA and specifically for SPPA, NFPPA and LPPA?; [2] What are the interventions with sufficient evidence of benefits?

Positive results were reported in all studies included in this review. It is important to mention that given the nature of the diseases no decline over variable periods can also be considered a positive outcome. Yet in order to be recommended these treatments require investigation regarding their efficacy. The approach characterized by randomized clinical trials is mandatory in the scientific literature pertaining to evidence-based medicine, but resorting to this approach is not always feasible when research addresses neuropsychological rehabilitation^50^ and, in particular, language and speech disorders.^51^ In the case of PPA, most studies consisted of single-case descriptions, which are very informative but do not allow for extensive generalization to other groups of patients. However, a large number of studies, combined with good study design, can help increase the treatments' level of evidence.

Results of the review of available scientific literature allows for recommendation of some nonpharmacological interventions for cognitive deficits following PPA. Using published criteria^8^ we recommend impairment-directed therapies aimed at naming and lexical retrieval in SPPA as Practice Options, based on 18 studies rated as Class III. For treatment aimed at object use, current evidence is based on two Class III studies. For functional interventions evidence is drawn from one Class III study. Therefore, more research is needed before we can reliably recommend these interventions.

Practice recommendations regarding interventions for NFPPA and LPPA are constrained by the small number of studies and patients that underwent nonpharmacological interventions reported to date.

Evidence for behavioral therapies aimed at improving typical deficits in NFPPA such as agrammatism, phonological skills and apraxia of speech is based on one study (for each deficit) rated as Class III^32,33,36^ respectively. Evidence from two Class III studies supports therapy targeting naming deficits^34,35^ and functional interventions using AAC^37,39^ in this group of patients.

The evidence for treatments targeting naming and spelling deficits in LPPA is based on two Class III studies^21,40^ and one Class III study,^41^ respectively.

One Class II study with PPA^48^ provides evidence that speech therapy can be beneficial for this group of patients compared to the condition of no treatment.

Generalization of gains has been observed in NFPPA and LPPA but there are very few reports to date. In SPPA, generalization to untreated items or functional communication situations has not been consistently reported. In general, better study design has been employed for impairment-directed interventions, however functional interventions have strong ecological validity and their gains should be investigated in further studies.

Post-treatment changes in brain activity have been addressed in only three studies and findings must be interpreted cautiously and replicated with comparable techniques and cognitive tasks. The current findings suggest that the brain plasticity mechanism engaged in therapy is reactivation and that patients recruit cortical areas that are typically preserved for the specific PPA variant to compensate for their dysfunctional language networks.

Concerning methodological issues, it is important that single-case studies include multiple baseline measures, treatment and control stimuli and/or treatment phases in which multiple measures are taken in the active phase of intervention and compared to measures when treatment is withdrawn. Long-term follow-up and measuring of treatment effects, as in the Henry et al.^43^ and Savage et al.^29^ studies, is also necessary. Controlled-group studies comparing interventions to placebo treatments are a challenge in this field but may be achieved with research collaborations (as in Farrajota et al.^48^).
