# Supplementary material for: Nonpharmacological interventions for cognitive impairments following primary progressive aphasia: a systematic review of the literature
Source: Dement Neuropsychol. 2013 Jan-Mar;7(1):122–31. doi: 10.1590/S1980-57642013DN70100018 (PMC5619554; doi:10.1590/S1980-57642013DN70100018)
Supplement: Supplementary file 1 [file dn-07-01-0122-suppl01.pdf]

APPENDIX 1

Table 1. Description of the variables analyzed in the intervention studies on Primary Progressive Aphasia - Semantic variant

**Studies uped by type ntervention**	**Characteristics of the participant(s)**<br><br>*Age (years), sex, education, disease duration*	**Intervention goals**	**Characteristics of the intervention**<br><br>*Duration; frequency and length of intervention*	**Materials**	**Procedures**	**Outcome measures**	**Main Results**	**Generalization**
**IMPAIRMENT-DIRECTED INTERVENTIONS**								
**Graham et al., 1999; 2001**	69; male; Doctor; 4 years	Naming and lexical retrieval	10 weeks; daily 30 min of controlled home practice (practiced items established by research team);<br><br>2 years; free daily home practice (as desired by patient)	lists of words and pictorial encyclopedias (e.g. Oxford English Picture Dictionary)	Repeated rehearsal of the name of concepts paired with pictures and real items, usually grouped by semantic categories.	Number of items generated in semantic verbal fluency tests including trained vs. untrained categories.<br><br>Baseline assessment, trained and control items, comparison to a cognitive unimpaired control group that did not undergo practice<br><br>10 week and 2 year follow-up	Better performance on semantic verbal fluency tasks involving categories that the patient underwent naming practice compared non-practiced categories.<br><br>Slower language deterioration compared to typical PPAS descriptions: no decline or even better performance in some language tests and verbal fluency tasks at follow-up	1) no generalization to semantic association tasks (i.e. Pyramids and Palm Trees Test)<br><br>2) Only trained items are retrieved in verbal fluency tasks
**Snowden & Neary, 2002**	61; female; N/A; N/A	Naming	Experiment I: 2 weeks; 1	booklet with pictures	Experiment I. Attempt to name the picture but not	Accuracy on trained items compared to	Proved ability to relearn vocabulary.	Naming performance deteriorated when

	54; female; N/A; N/A		session/week<br><br>Experiment II: 20 days; 20min of daily home practice		to guess followed by its presentation in written and spoken forms. Patient asked to read aloud written word matched to picture (errorless learning strategy)<br><br>Experiment II. One day training with therapist in which naming was trained as in experiment I added to definition and meaningful context association cues. Self-study phase: patient got a booklet with test materials for 20 days. Naming and personalized contextual cues were practiced with the help of spouse.	baseline assessment. Reverse-order control.<br><br>Follow-up:<br><br>2 week- and 4 month- (experiment I)<br><br>6 month- (experiment II)	Experiment I: mild improvement in naming, better for pictures that patient retained semantic knowledge<br><br>After 4 months, naming declines but not to baseline level.<br><br>Experiment II: relearning of items for which patient retained no semantic knowledge before training in the contextualized cued condition. Maintenance of gains after 2 months and decline, but not to baseline levels after 6 months.	test items were presented out of context (i.e. cards instead of booklet) and in randomized order, but not in the reverse order condition.
**Bozeat, Patterson & Hodges, 2004**	58; female; N/A; 3 years	Object use	1 single session	Objects and their respective recipients	Following each object display, the patient was given the object and its recipient and asked to copy the movement made by the examiner. The objects in the	Accuracy of ability to use target trained objects vs. control set.<br><br>Baseline assessment, after-training retest,	Patient relearned how to manipulate objects through observation of their use, despite being at advanced stage of PPAS.<br><br>Maintenance of gains	There was a small increase in performance over time on the untrained objects

					control set were simply given to the patient, along with their recipients, and patient was asked again to demonstrate the use of each.	same day retest<br><br>Follow-up: one and five weeks	over five weeks.	
**Frattali, 2004**	66; male; higher education; N/A	Naming	3 months; 1 session of 2 hours/week	Picture cards	A set of cards within a semantic category was presented and patient and experimenter engaged in a conversational interchange, focused on associative and analytical skills (effortful and errorless learning strategy). Patient discouraged to name items. No explicit reinforcement was given when patient occasionally named it. After each set, cards were shown again and if the name of it came to the patient, he was allowed to say it.	Naming accuracy on treated vs. untreated items. ABA design. Multiple baseline assessment, randomized order of presentation of stimuli and semantic categories across trials.<br><br>Follow-up: 3 month.	Significant improvement in naming during the active phases of treatment.<br><br>No maintenance of gains at follow-up.	No generalization of naming ability to novel stimuli.
**Jokel, Rochon & Leonard,**	63; female; Bachelor; 7 years	Naming	3 weeks; daily home practice;	Picture cards with name and	Picture naming (looking at the picture and reading	Naming accuracy on treated vs.	Better performance on naming treated words and	Not achieved

**2002; 2006**			30 min/day	definition on the back	aloud name/definition)	untreated items.<br><br>Multiple baseline assessment, trained and control items,<br><br>Follow-up: one and six months	maintenance of vocabulary trained. Decline on untrained words.	
**Bier et al., 2009**	70; female; High school; 5 years	Concept relearning (Naming and semantic attributes)	3 months, 12 sessions	Pictures and names belonging to four different semantic categories	Formal-semantic therapy and spaced-retrieval method: picture was presented along with its spoken name, specific attribute and written name of the category twice; pictures presented again with increasing time-recall intervals and patient was asked to name them and generate semantic attributes. Semantic feedback and cueing technique was used when patient was unable to name items.<br><br>Simple repetition method was added to procedures above in half of sessions.	Accuracy in naming and generation of general and specific semantic attributes<br><br>Multiple-baseline assessment.	Significant improvement in naming and learning specific semantic attributes of trained items.<br><br>No advantage observed for the spaced-retrieval condition compared to repetition<br><br>Maintenance of gains at follow-up of 5 weeks	Not achieved for untrained categories

**Dewar et al., 2009**	63; male; Bachelor; 4 years	Naming and learning semantic attributes	5 sessions (total)	Black and white photographs of famous people in UK	Combination of three methods: mnemonic, vanishing cues and expanded rehearsal. Photograph was first presented, paired with its name and semantic fact, to form a mnemonic of it. Then, photograph was presented with written name and semantic fact, but words and letters were removed little by little, and patient had to repeat information with less cues than at the preceding point, till the moment when patient could state the name and fact without any cueing. Consolidation of this learning was undertaken with expanded rehearsal.	Baseline assessment (free recall and free plus cued recall measures)<br><br>Naming and recalling of the semantic fact accuracy on treated vs. untreated photographs.<br><br>Reverse-order control.<br><br>Follow-up: 2-week.	Five training sessions improved naming without cues in virtually all stimuli names. Cued recall was not necessary to boost the performance of the patient.<br><br>Maintenance of practiced and non-practiced items even after 2 weeks by free recall.	Generalization of naming to different photographs of the same famous people was observed.<br><br>No influence of a change in order was observed.
**Heredia et al. 2009**	53; female; well-educated civil servant; 2 years	Naming	Trained daily at home for a month	Power-point presentation of 28 items. Items were selected from	Presentation of a picture on a computer screen for naming followed by the same picture with	Naming accuracy on trained vs. control items; report of carer about use of trained items in	Improved performance on trained items and maintenance after 6 months	Not achieved for untrained items.<br><br>Anedoctal report of

				pictures patient failed to name in the language assessment.	correspondent written name to read aloud.	conversation,<br><br>Follow-up: one and six month.		generalization to spontaneous speech.
**Newhart et al., 2009**	60; female; Master; N/A	Naming and lexical retrieval	8-9 weeks; approx. 2-3 sessions of 30-60min/week, total of 29 sessions	Picture cards of objects (of 3 semantic categories) and notebook with the written name of all items organized by category	Cueing hierarchy treatment: 1) spontaneous oral naming of the pictured object; 2) written naming and oral reading of it, 3) notebook search, reading aloud the written name; 4) repetition of reading aloud the written word; 5) repeat the name of the pictured object. 6) Then, all the steps backwards.	Multiple baseline assessment.<br><br>Naming accuracy on Trained vs. untrained items, pre and post test assessment.	Temporary improvement of naming on treated items.<br><br>Less deterioration in untreated items from treated categories (when compared to untreated items of untreated categories)	Not achieved for untrained items.
**Robinson et al., 2009**	63; female; some college; 3 years<br><br>63; female; N/A; 3 years	Naming, definition and object use	3 weeks; 2 sessions/week. Home practice was emphasized.	Kitchen utensils, stationary items, tools and their respective utensils. Video for self-modeling.	Errorless learning strategy: 1. Object was presented with its name and description. Patient was prompted to repeat it. 2. Object and its recipient were shown and its name was said. Patient was prompted to repeat the name again. 3. A personally relevant definition	Baseline assessment: Performance in naming, definition and object use before and after therapy were measured. Familiarity with objects was controlled. Matched control group. Randomized order of presentation of	Both patients produced significantly more definitions rated as “good” for trained and untrained objects (post-therapy), and improved some aspects of object use.<br><br>Improvement for untrained objects was not evident at follow-up.	Not clear generalization of this therapy to everyday use .

					to the object was provided. Patient repeated the definition. 4. Demonstration of use was provided, and the patient was prompted to replicate the actions.	stimuli. Trained and untrained items.<br><br>Follow-up: 1-month.	Only one of the patients had significant improvements in naming. The same patient maintained therapy gains over time, but the other declined for definitions and some aspects of object use (but still remained above the baseline)	
**Dressel et al., 2010**	48; male; college; 2 years	Naming	4 weeks; 5 sessions/week	Picture cards with line drawings presented on a computer screen	A picture was presented and patient should name it, assisted by a hierarchy of oral cues. Cues were either phonological or semantic, composing the lexical-phonological and lexical-semantic access training	Accuracy on naming treated and untreated items. Error pattern analysis (errors were coded as semantic, phonological, mixed, unrelated, etc.).<br><br>Multiple baseline assessment with naming tests, one pre-test and both post-tests were made inside the MR scanner.<br><br>Follow-up: 2-month.	Cueing hierarchies were successful to achieve immediate training effects.<br><br>Most common error types were omission, semantic paraphasias and mixed errors.<br><br>fMRI data informed that improved naming following therapy was also mirrored by changes in cortical activities (right superior and inferior temporal gyrus) – authors interpreted it as compensatory mechanisms of the RH.<br><br>Maintenance of therapy gains was	No significant generalization to control items.

							limited.	
**Jokel, Rochon & Anderson, 2010**	N/A; male; bachelor; 2 years	Naming	4 months; 3 sessions of 60min/week	MossTalk words computer program	Picture was presented on computer screen, a software-generated spoken definition and written word was provided. Patient looked at the picture, listened to the description and then repeated the word.	Accuracy on treated vs. untreated items.<br><br>Post-test checking generalization to different contexts (language battery of tests), connected speech and quality of life<br><br>Multiple baseline across behaviors design.<br><br>Follow-up at 1 and 3 months later	Better performance on treated words vs. untreated.<br><br>Maintenance of vocabulary trained but decline on untrained.<br><br>Better performance and maintenance for items that patients retained some semantic knowledge.	Patient could name trained items in different contexts (better performance in a naming test that included some items that were practiced in the computer-based therapy).<br><br>Retrieved more items in animal verbal fluency test but did not reach statistical significance.<br><br>No significant improvement in quality of life or connected speech measures, although scores were a bit higher after treatment.
**Montagut et al., 2010**	68; male; elementary; 7 years	Naming and lexical retrieval	5 weeks; 4 sessions of 15min/week	Picture cards of frequent use (of 5 semantic categories)	Picture naming without cues first and, if necessary, written name matched with picture.	Accuracy on trained items compared to baseline assessment.<br><br>Follow-up: one and six month.	Improvement of naming on treated items.<br><br>After 1 and 6 months, naming declines progressively, but not to baseline level.	Generalization of naming ability to another picture of the same concept.

**Senaha, Brucki, Nitrini, 2010**	55; female; some college; 2 years<br><br>77; male; bachelor; 1 year<br><br>56; male; bachelor; 2 years	Naming and lexical retrieval	6-18 months; 1-2 sessions/weeks	Picture cards, photographs and written descriptions	Naming pictures, photographs and descriptions. With progression in correct answers in naming, cues were gradually removed	Naming accuracy on treated vs. untreated items, pre and post test assessment.<br><br>Baseline assessment.	Improvement in naming treated items, in spite of decline in performance on untreated items	No generalization was achieved.
**Mayberry et al., 2011**	65; female; N/A; 4 years<br><br>53; male; N/A; 4,5 years	Naming	3 weeks; daily training at home In the first session researcher introduced training procedures, other sessions were carried out over the phone	A booklet containing picture stimuli to be trained	Picture naming without cues first. Then, turning page and reading aloud written name matched with picture.	Naming accuracy of trained vs. untrained items; baseline, pre and post treatment assessments.<br><br>Analyses of naming errors to study learning mechanisms (errorful learning)<br><br>Follow-up: 1 month	All items learned by the two patients and total maintenance of gains after a month without training	There was limited, but appropriate generalization to untrained items in one case and this was maintained at follow-up.<br><br>Overgeneralization (making use of trained labels incorrectly) was observed in the two patients, particularly for foils which were visually and semantically related to the treated items.
**Jokel & Anderson, 2012**	From 56 to 87; 3 males and 4 females; from high school to master degree; from 2 to 6 years	Naming	8-12 weeks; 2 to 3 sessions of 60 minutes per week	Sets of pictures that patients failed to name on baseline.<br><br>50% of the	Four types of treatment were compared:<br><br>Errorless passive picture is shown on computer screen and semantic and	Naming accuracy of treated and untreated items pre and post treatment; Effect sizes calculated	Naming accuracy improved for trained items.<br><br>Errorless learning techniques lead to	Patients could name trained items in different contexts (better performance in a naming test that included some items that were

				pictures participants were unable to name and comprehend.	phonemic cues are provided;<br><br>Errorless active: picture is shown and questions about it are made;<br><br>Errorful passive: picture is shown, wrong semantic and phonological cues are provided, patient is asked to guess;<br><br>Errorful active: picture is shown, open questions about item are made.	Follow-up at 1 and 3 months	better results than errorful ones. No difference between active or passive learning.<br><br>Better results on items for which patients retained semantic knowledge.	practiced).<br><br>Retrieved significantly more items in animal verbal fluency test.<br><br>No significant improvement in quality of life or connected speech measures, although scores were a bit higher after treatment.<br><br>Three participants reported use of learned words in functional communication situations, but this was not formally tested.
**Savage et al., 2012**	From 54 to 69; 4 males; some college; from 4 to 5 years	Naming and lexical retrieval	3-6 weeks; 30-60min of daily home practice	Digital pictures of individual's household objects, lists of names and definitions of objects, recordings of definitions and names provided by	Study I: Word-picture matching training program: 1) Picture of item was shown on screen, patient was asked to recall (but not to guess) the name; 2) Picture and word paired were shown on screen, patient read it aloud; 3) Written form and audio	Multiple baseline approach with three word lists.<br><br>Naming accuracy, with treated vs. untreated items (presented in a randomized order); effect sizes calculated<br><br>Follow-up: 2-, 3-	Better performance on ability to name trained items, and no changes in untrained items.<br><br>Large effect sizes measured to patients with severe deficits. Medium/small effect sizes for the milder patient.<br><br>High levels of	No generalization reported.

				the patient. (Used items were at least partially still meaningful to patients)	recording of item description provided by the patient were shown, and patient listened to it; 4) Word and picture paired disappeared and patient concentrated on the word; 5) Word and picture reappeared, and voice recorded name of item was played, patient repeats it aloud.<br><br>Study II: Included a semantic description task to the prior study.	and 6-week.	achievement produced within a 3-week period.<br><br>Items trained for longer periods (six weeks) showed better maintenance than items trained for three weeks.<br><br>No need to provide definitions or ask for sentence generation to achieve same levels of improvement.	
**FUNCTIONAL INTERVENTION**								
**Wong et al., 2009**	63; male; 14 years; 2 years	Communication effectiveness: improvement/ maintenance of discursive skills	4 years of intervention<br><br>Combined intervention (group and individual sessions and carer trained to use intervention principles in communication activities with the patient)	pictures, flyers, newspaper articles, books, personal photographs	Individual and group sessions. Client-focused discourse intervention to develop turn-taking awareness, combined use of verbal and nonverbal modes of expression and communicative functions. Facilitation of communication	Scale of communication effectiveness, pre-test and two years later post-test.	Therapy results are of difficult interpretation due to patient's language decline over the years and absence of a control group or measure.	Not reported in the study

					effectiveness with education and training of the care-giver. Themes included: social exchange, discussion of current events, discussion of informational topic and discussion of life story events.			
**Bier et al., 2011**	68; female; bachelor; 4 years	Learning semantic attributes/ activity participation rehabilitation	5 months; 1 session biweekly	Kitchen with ingredients and utensils; a computer program called SemAssist, which provided the steps to prepare the target recipe; a target recipe (shrimp); other three control/ neutrals recipes.	Ecological rehabilitation. Practicing cooking a target recipe many times. SemAssist software was installed in a notebook in patient's kitchen, and was used whenever she wanted to provide her with the steps of the activity (cueing). Experimenter measured generation of semantic attributes of ingredients found in the target recipe while cooking, time taken during preparation, type of errors, frequency of use of SemAssist, etc.	Multiple baseline assessment (ABA); Target vs. control and neutral recipes comparison.<br><br>Follow-up: 2- and 6-month.	The number of errors and cues needed during food preparation decreased with therapy. With the use of SemAssist, patient was able to prepare recipes on her own.<br><br>Generation of semantic attributes of ingredients of cooked meals increased compared to control recipes.<br><br>Therapy led patient to spontaneously cook again; but cooked less often at post-measures.	Not achieved.<br><br>Abilities and knowledge were very specific to the practiced context.<br><br>Naming abilities were tested and did not improve, even for pictures of ingredients found in the recipes.

Table 2. Description of the variables analyzed in the intervention studies on Primary Progressive Aphasia - Nonfluent/Agrammatic variant

**Studies grouped by type of intervention**	**Characteristics of the participant(s)** *Age (years), sex, education, disease duration*	**Intervention goals**	**Characteristics of the intervention** *Duration; frequency and length of intervention*	**Materials**	**Procedures**	**Outcome measures**	**Main Results**	**Generalization**
**IMPAIRMENT-DIRECTED INTERVENTIONS**								
**Schneider, Thompson & Luring, 1996**	62; female; some college; 2,5 years	Agrammatism	16 sessions (total)	Drawings and videotapes showing gestures to facilitate sentence production	Target picture placed in front of the patient, demonstration of a gesture in video and production of a sentence combined with gesture.<br><br>Pre-training of gestures at home before treatment.	Accuracy in oral and gesture production of verb tenses in sentences.<br><br>Narrative discourse production compared to controls.<br><br>Multiple baseline across behaviors.<br><br>Follow-up: 3 months after treatment	Improved production of sentences on trained items and untrained verbs within trained tenses.<br><br>In the follow up: gestural responses were maintained at criterion while verbal responses declined for future and pasts tenses.	Generalization to untrained items
**Louis et al, 2001**	64; female; N/A; N/A<br><br>71; female; N/A; N/A	Phonologic skills	42 days of daily 15-20min home practice.<br><br>One patient	Audio CD with auditory exercises tapping syllabic and	Auditory exercises based on the "odd-one-out model". For facilitation and improvement of	Baseline assessment; mean performance on trained tasks was calculated.	Significant improvement in fluency in 1 case; written comprehension in	Patients demonstrated improvement in tasks involving oral expression

	77; male; N/A; N/A		received additional 60-day treatment	phonemic segmentation;	auditory perception, signal of speech was slowed by 166% in the exercises.	Pre and post-test assessment with Boston Diagnostic Aphasia Examination	1 case; repetition in 2 cases; reading in 2 cases. No improvement in neither naming nor oral comprehension. Number of phonemic paraphrasias reduced in one case, and unchanged in two.	although the training focused auditory skills.
**Jokel et al., 2008**	58; female; bachelor; N/A<br><br>75; female; bachelor; N/A	Naming and lexical retrieval	2-4weeks; 2-3 sessions of 1hours/week	Digital pictures, MossTalk Words® (computer-based treatment for anomia)	Picture naming. Presentation of a cueing hierarchy consisting of written initial letter, written whole word cues, and repetition (when necessary) .	Multiple baseline assessment.<br><br>Accuracy in naming.<br><br>Follow-up: 6-month after	Improvement in naming skills.<br><br>Score returned to the baseline after 6 months	Generalization to untreated items did not occur<br><br>Some generalization of therapy effects was seen at the syntactic level immediately after treatment.
**Marcotte & Ansaldo, 2010**	60; male; Professional; 2 years	Naming (nouns and verbs)	3 weeks; 3 sessions of 60min/week	Picture cards and objects	SFA – Semantic Feature Analysis therapy.<br>Picture naming. When unable to name it, semantic features were prompted	Accuracy in naming tasks. Event-related fMRI measures during oral picture naming.<br>Comparison to a patient with stroke.	Improved naming performance.<br><br>Activation of areas related with semantic processing, especially the left middle and superior temporal gyrus and	Not evaluated

							bilateral inferior parietal lobe for nouns and left inferior and middle temporal gyrus for verbs Recruitment of larger networks (more voxels activated) compared to the stroke patient.	
**Henry et al. 2013**	73; female; Professional; 5 years	Speech production (apraxia of speech)	12 weeks; 1 session of 1 hour/week Oral reading practice at home was advised	Paragraphs with multisyllabic words included	Structural oral reading of texts: Patient reads aloud a randomly-selected paragraph from the previously given homework to have pronunciation of words therein assessed. Therapists corrects major speech sound errors or incomplete productions in multisyllabic words	Baseline assessment.<br><br>Accuracy in the production of multisyllabic words<br><br>follow-up: 3- and 6-month and 1-year.	Multisyllabic word production in trained text became more accurate and stable over the course of treatment and was error-free over the last four treatment sessions.	Self-ratings of treatment effects indicated perceived improved performance in reading aloud and speaking (fluency) as well as improved confidence and reduced frustration in communication with familiar and unfamiliar people.
**FUNCTIONAL INTERVENTIONS**								

**Murray, 1998**	64; female; high school; 4 years	Auditory and reading skills/ Communicative skills	2,5 years, three treatment phases (T1, T2,T3)<br><br>T1: twice a week, 45 minutes, total 46,5 h<br><br>T2:individual treatment<br><br>T3: 41 h of individual treatment + 10 h of group sessions	T1:Oral and Written paragraphs<br><br>T2: drawing board<br><br>T3: AAC device	T1: Traditional stimulation, facilitation approach Reading or listening to a paragraph then answering orally or in written form to yes/no questions or questions that required responses of two to four words<br><br>T2: Back to the drawing board program – train the use of drawings to improve functional communication<br><br>T3: Functional communication approach including AAC device Patient tries to speak after therapist and feedback is provided	T1:Accuracy on trained and untrained language tasks during active phase of training and comparison to no-treatment phases<br><br>T2: pre and post treatment evaluation including accuracy in producing drawings<br><br>T3:Qualitative analysis comparing pre- and post-treatment conversational interactions	T1: improvement on practiced tasks<br><br>T2: drawings depicted main events more accurately and had greater detail and clarity<br><br>T3: patient and her spouse increased their repertoire and use of repair strategies	T1: no generalization to functional communication<br><br>T2: poor generalization to functional communication, although patient started to mix drawing and writing to answer questions<br><br>T3: does not apply
**Rogers & Alarcon, 1999**	69; male; Master; 4 years	Communicative skills	4 years	AAC device, paper, pen, etc.	Activity-participation focus led to increasing use of AAC including gesture, writing, drawing, partner training, communication book, AAC device.	Not mentioned	Not mentioned	Not mentioned

**Pattee C, Von Berg S, Ghezzi P. (2006)**	57; female; N/A; 5 years	Communicative skills	9 weeks; 8 sessions	Colored cards of photographs depicting daily activities	Patient describes the activities depicted in the photographs using two modes:<br>1) American sign language (ASL)<br>2) Alternative Communication text-to-speech device (ACD)	Baseline assessment<br><br>No follow up	Increases in content information across all measures, but more so for ASL.	No generalization task was tested.

Table 3. Description of the variables analyzed in the intervention studies on Primary Progressive Aphasia - Logopenic variant

**Studies grouped by type of intervention**	**Characteristics of the participant(s)** *Age (years), sex, education, disease duration*	**Intervention goals**	**Characteristics of the intervention** *Duration; frequency and length of intervention*	**Materials**	**Procedures**	**Outcome measures**	**Main Results**	**Generalization**
**IMPAIRMENT-DIRECTED INTERVENTIONS**								
**Newhart et al., 2009**	65; female; Master; N/A	Naming and lexical retrieval	8-9 weeks; approx. 2-3 sessions of 30-60min/week, total of 24 sessions	Picture cards of objects (of 3 semantic categories) and notebook with the written name of all items organized by category	Cueing hierarchy treatment: 1) spontaneous oral naming of the pictured object; 2) written naming and oral reading of it, 3) notebook search, reading aloud the written name; 4) repetition of reading aloud the written word;	Multiple baseline assessment<br><br>Nming Accuracy in trained vs. untrained items, pre and post test assessment.	Improvement in naming of treated items and untreated items in both treated and untreated categories	Generalization to untreated items in treated categories as well as untreated items in untreated categories

					5) repeat the name of the pictured object. 6) Then, all the steps backwards.			
**Beeson et al., 2011**	77; male; professional; 2,5 years	Naming and lexical retrieval	2 weeks; 6 sessions of 2 hours/week + 1 hour of daily home practice	Colored photographs cards with removable written labels	Repeated retrieval of words from a semantic category under a time constraint	Generative naming performance<br><br>Multiple baseline assessment trained and control items<br><br>Follow-up: 3 weeks, 4 and 6 months after	Improvement of ability to retrieve exemplars for the trained and untrained categories.<br><br>Performance on the trained categories was significantly better than pre-treatment performance at the 6- month follow-up<br><br>Changes in post-treatment fMRI activation suggested the behavioral improvements were supported by increased reliance on left prefrontal cortex during word retrieval	There were improvement in discourse efficiency, generalization of the semantic elaboration and self-cueing strategies
**Tsapkini & Hillis, 2013**	62; female; bachelor; 6 years	Spelling	11 weeks; 1 session of 1-2hours/week	Paper, pen, lists of English most-	learning of phoneme-to-grapheme correspondences with help from key words	Baseline assessment, trained and untrained items.	Patient was able to learn associations between	Generalization to other untrained tasks post

				common word initial phonemes and words starting with that sound		No follow-up for the PPA patient	phonemes and graphemes as well as between phonemes and words.<br><br>No difference between treated and untreated items.<br><br>Generalization to other untrained tasks such as pointing to named letters	intervention

Table 4. Description of the variables analyzed in the intervention studies on Primary Progressive Aphasia (general)

**Studies grouped by type of intervention**	**Characteristics of the participant(s) and further information on PPA**	**Intervention goals**	**Characteristics of the intervention** *Duration; frequency and length of intervention*	**Materials**	**Procedures**	**Outcome measures**	**Main Results**	**Generalization**
**IMPAIRMENT-DIRECTED INTERVENTIONS**								
**McNeil, Small, Masterson, Fossett, 1995**	61; male; N/A; 9 months (no further information about the patient's	Lexical semantic retrieval (adjectives, prepositions and verbs)	5 months; approx. 2hours/week (1 hour of treatment and 1 of baseline probes collection)	Lists of words (nouns were excluded) grouped as antonyms and synonyms	Orally presenting to the patient a series of lexical items from a list of 10 predicative adjectives and	Multiple baseline. Accuracy on trained items compared to baseline assessment. Scores improved beyond baseline in	Extended practice of treatments were effective for both antonym and synonym adjectives.	Generalization to untrained adjectives, verbs and prepositions was observed.

	impairment was given)				asking him to vocally produce either antonyms or synonyms for the word or phrase. Cueing (hierarchically provided) was added after an error. Correct answers were verbally reinforced. Treatment combined phases of only behavioral with behavioral and pharmacological (dextroamphetamin e), manipulations.	the antonym task	Only behavioral treatment phase was compared to combined behavioral and pharmacological phase and there were "essentially equivalent results".<br><br>Maintenance was initially high (1 month) for all treated and probe lists, but performance approached baseline around 3,5 months after treatment.	No effects could be noticed regarding subject's discourse level.
**Finocchiaro et al., 2006**	60; male; N/A; N/A	Naming and lexical retrieval	75 days; 5 sessions/week with 15 days of intervals	hf-rTMS equipment	10 consecutive stimulations of 40 pulses each, followed by a 30-sec pause	Baseline assessments, use of a controlled condition for comparison	Improved performance in verb production and not in other tasks	Difficult to rule out.<br>Some limitations, for instance performance in tasks with nouns was at ceiling in baseline
**Henry, Beeson & Rapcsak, 2008**	N/A; N/A; N/A; 5 years (fluent with characteristics towards non-fluent aphasia,	Naming and lexical retrieval	16 days; daily sessions of 90min + home practice	Cards with pictures and words, picture dictionary organized thematically	Guided lexical retrieval prompted by identification and elaboration of items within subcategories, and	Multiple baseline assessment, trained and untrained categories. Randomization of presentation order of	Improved lexical retrieval was observed on the generative naming task for trained categories	No generalization for untrained items.

	incl. mild agrammatism, phonemic paraphasias, and apraxia of speech)<br><br>N/A; N/A; N/A; 6 years (fluent aphasia, surface dysgraphia)			by category	other semantic tasks (sorting pictures and words by subcategory, identifying semantic attributes of exemplars, and picture naming using a picture dictionary)	stimuli. Effect size calculation. Comparison between subjects (and with a stroke case).<br><br>Follow-up: 3-week and 4-month, for one of the cases	Study has accounted for effect size, but noted a very small effect on the treated list.<br>The one patient who took follow-up probes maintained improved performance.	
**Rapp & Glucroft, 2009**	55; female; college; 9 years (dysgraphia)	Spelling	15 weeks; biweekly 1,5 hour sessions; homework assignments.	Notecards with written words	Spell-study-spell intervention: 1) To spell each word to dictation, 2) to study the word presented on a written notecard while the experimenter repeated the word and orally named each of its letters, and if the word was spelled incorrectly, 3) to attempt to spell it again.	Baseline assessment, accuracy on trained vs. control words, 6- and 12-month follow-up	Significant improvement of trained words compared to baseline;<br><br>Advantage in accuracy of trained words over control homework and repeated word sets;<br><br>Accuracy on trained words persisted over control words in 6-month follow-up.<br><br>At 12-month follow-up, all word sets had significantly	No generalization effects documented

							declined to baseline.	
**Snowden et al. 2012**	60; male; Academic; 2 years	Facilitating access to letter names and sounds (to assist reading words aloud)	12 months; daily home practice	Pictures and word cards, country name lists associated with each written letter of the alphabet	Written letters of the alphabet were associated with country names. Consonants trained before vowels (according to patient's performance in baseline assessment)<br><br>When patient could not retrieve a written letter sound, he spoke the associated country name thus providing him with the letter sound. An associative strategy by which letter names were linked to concrete words and pictures was also adopted to teach him letter names.	Accuracy in the ability to name and sound letters as assessed by Palpa letter naming/sounding test.<br><br>Ability to generate country names associates.<br><br>Baseline assessment, two assessments during intervention (6 and 12 months) and follow-up 2 and 3 years later without further training	Improvement on treated but not untreated letters (consonants vs vowels according to the phase of treatment)<br><br>Letter naming and sounding performance declined to 0% two years after therapy completion.<br><br>Ability to generate country's names in response to a letter learnt in language therapy remained relatively robust 3 years after therapy.	The semantic strategy used in therapy was employed functionally to write object names in the period patient was participating in therapy.
**FUNCTIONAL INTERVENTIONS**								
**Cress and King, 1999**	59; female; N/A; 5 years<br><br>60; male; doctor; 7 years.	Communication, comprehension and expression	Case 1: 3 sessions<br><br>Case 2: 1 month; 3-4 sessions of 1 hour/week. Home	Case 1: Communication book, cards with pictures and names	Several specific intervention techniques.<br>Case 1 used writing skills to	Case 1: subjective analysis from the therapist point-of-view.	Case 1 e 2: therapy was described generally as a positive	There was improvement in the communication, but it was not

	For both cases, MRI revealed atrophy of the left temporal lobe, and got the diagnosis of PPA without dementia.		practice with his relatives involved in therapy	Case 2: AAC boards	communicate and graphical images, such as maps to communicate with therapist, for instance.<br><br>Case 2: Training with the AAC pictures.	Case 2: qualitative analysis of conversation samples recorded at home by "communication partners"	experience. No specific control and measures were mentioned.	described about generalization for untrained items
**Cartwright & Elliott, 2009**	From 59 to 66; tertiary education; 4 PPA (3 nonfluent aphasic women, 1 man with dense semantic deficits); N/A	Enhancing participant's access to TV content	10 weeks including pre and post-test sessions; 90 minute-group weekly sessions plus two additional practices in the same week with care-giver at home	Glossary of vocabulary items commonly used within the genre of television viewing, 10 episodes of Australian story series, whiteboard where plot information was written/drawn available to participants throughout the session, feature analysis guide sheet providing each participant with accessible summary of	Participants viewed a total of 10 novel episodes of the Australian story series over a total of 10 weeks. Strategies to facilitate comprehension and recall of episode content were applied. Care-giver trained to assist patient at home.	Number of episode story information units in spoken discourse;<br><br>Transactional success (discourse analysis undertook by an independent listener);<br><br>Discourse comprehension questions<br><br>Pre and post intervention assessments and comparison to a control group of cognitive unimpaired individuals in the first measure.<br><br>Control group was not exposed to treatment only pre and post-test assessments.	Higher number of episode story information units in spoken discourse post treatment, close to control level; more story information units conveyed to a naive non-expert listener (transactional success), improved ability to answer discourse comprehension questions in one subject (patient with semantic deficits).	Not reported

				episode content.				
**Farrajota et al., 2012**	68 (aprox.); N/A; 11,6 years (aprox.); N/A<br><br>2 PNFA, 2 SD, 6 LPA (10 patients analysed as group)	Improvement of the patient`s ability to communicate by verbal means in everyday life situations	11 months; 1 session of 1hour/week, approximately 38 sessions	Pictures and materials for multimodal stimulation	Schuell's multimodality stimulation approach – includes picture naming, descriptions of picture actions, complex auditory-verbal comprehension, reading and writing, facilitation of expression of feelings and opinions, and enhancement of conversational skills	Accuracy in Snodgrass and Vanderwart naming test (primary) plus other language measures testing auditory comprehension, repetition and object identification (secondary)<br><br>Patients were assessed before and after intervention and their performance compared to a PPA no-speech therapy control group matched for age, education and language deterioration but not by PPA subtype.	Significant main effect of therapy was found in the naming test when compared to control group.<br><br>No difference in other language measures.	Not reported in the study.
